# The Effects of Obesity, Six Weeks of Aerobic Training, and Cold Water Exposure on the Expression of FNDC5 and UCP1 Genes in Male Wistar Rats

**DOI:** 10.5812/ijem-142746

**Published:** 2024-10-29

**Authors:** Sadegh Tohidi, Seyyed Reza Attarzadeh Hosseini, Mohammad Mosaferi Ziaaldini

**Affiliations:** 1 Ferdowsi University of Mashhad, Mashhad, Iran; 2 Faculty of Sports Sciences, Ferdowsi University of Mashhad, Mashhad, Iran; 3 Sport Physiology Department, Ferdowsi University of Mashhad, Mashhad, Iran

**Keywords:** Obesity, Exercise, Cold Temperature, Thermogenesis, FNDC5, UC51, Subcutaneous Fat, Adipose Tissue, White

## Abstract

**Background:**

Obesity is a complex disease that has become increasingly prevalent. While obesity itself is not new, its widespread occurrence is a more recent concern. Stimulating brown adipose tissue (BAT) and promoting the browning of white adipose tissue (bWAT) have shown promise as therapeutic targets to increase energy expenditure and counteract weight gain.

**Objectives:**

This study aimed to investigate two main aspects. First, we examined how obesity affects the expression of the fibronectin type-III domain containing 5 (FNDC5) and uncoupling protein 1 (UCP1) genes in male Wistar rats. Second, we assessed the effects of six weeks of aerobic exercise, exposure to cold water, and the combination of both on the expression of the FNDC5 and UCP1 genes in obese male Wistar rats.

**Methods:**

In this experiment, 25 male Wistar rats were randomly assigned to five groups (5 rats per group) after inducing obesity. The groups included: A control group (C), an obesity group (O), an obesity group exposed to cold water (OC), an obesity group engaged in aerobic exercise (OE), and an obesity group exposed to both cold water and aerobic exercise (OCE). The aerobic exercise sessions lasted 30 - 60 minutes, with a speed of 15 - 25 meters per minute. The cold water exposure protocol involved shallow water (2 - 4 cm) with a temperature of 14 - 18°C. The OCE group performed both aerobic and cold water exercises in each session. The expression of the FNDC5 gene in the soleus muscle and the FNDC5 and UCP1 genes in subcutaneous fat was evaluated using Real-Time PCR. All statistical analyses were performed using SPSS software version 16, with a significance level set at P ≤ 0.05.

**Results:**

Obesity significantly increased the expression of the FNDC5 gene (P = 0.008). After six weeks of aerobic exercise (P = 0.016) or cold water exposure (P = 0.016), there was a significant decrease in FNDC5 gene expression. Surprisingly, the combination of both interventions did not result in a significant effect (P = 0.75). On the other hand, none of the interventions—whether aerobic exercise, cold water exposure, or their combination—had a significant effect on the expression of the UCP1 gene (P > 0.05).

**Conclusions:**

The increase in FNDC5 gene expression caused by obesity may serve as a compensatory mechanism to cope with the condition. However, both cold water exposure and aerobic exercise appear to mitigate this increase in FNDC5 gene expression through enhanced thermogenesis.

## 1. Background

Obesity results from an imbalance between energy intake and energy expenditure, making it a chronic disease. It is projected that by 2030, 51% of the population will be affected by obesity, highlighting the urgent need to explore effective strategies and safe medications (1). In today's society, consistently reducing calorie intake is a challenging task for obese individuals. Therefore, thermogenesis, which increases energy expenditure, has emerged as a potential solution to combat obesity. Mammalian adipose tissue consists of two distinct types: White adipose tissue (WAT) and brown adipose tissue (BAT). White adipose cells are characterized by a large lipid droplet at their center and relatively fewer mitochondria compared to other cell types. In contrast, BAT is primarily responsible for generating heat and contains small lipid droplets along with a significant number of mitochondria (2).

Thermogenesis refers to the physiological process of generating additional heat beyond the basic metabolic rate by utilizing stored chemical energy (3). This process can be divided into two subcategories: Shivering and non-shivering thermogenesis (NST). Non-shivering thermogenesis primarily occurs through metabolic activity in BAT and, to a lesser extent, in skeletal muscle, liver, brain, and WAT (4). In rodents, exposure to cold is known to be the strongest non-shivering thermogenic stimulus (5). When sympathetic nerves are stimulated during exercise or cold exposure, WAT can undergo a transformation into beige adipocytes, which possess thermogenic properties similar to those of BAT.

The increased thermogenesis observed in skeletal muscle is associated with a specific protein called fibronectin type-III domain containing 5 (FNDC5) (6). Fibronectin type-III domain containing 5 can be cleaved, leading to the production of a secreted muscle factor known as irisin. Irisin acts as a crucial myokine that influences subcutaneous WAT through the bloodstream, promoting thermogenesis by increasing the expression of uncoupling protein 1 (UCP1) (2, 6).

On the other hand, shivering thermogenesis occurs through the rhythmic contraction and relaxation of skeletal muscles. This process induces FNDC5 expression and irisin secretion at levels comparable to those observed during exercise, suggesting that shivering links exercise and cold-induced thermogenesis (7). Prior research on the association between obesity and the browning of white adipose tissue (bWAT) has yielded contradictory results. Li et al. documented an inverse correlation (8-10), whereas Guilford et al. observed a direct correlation (11-13), and Badawy et al. found no substantial association (14, 15) between body fat mass and bWAT. As a result, further research is needed to better understand the role of bWAT in obesity. It remains unclear whether the increased expression of these genes following obesity contributes to energy expenditure or if this increase itself is a pathological factor. Consequently, individuals with obesity may not experience the same metabolic improvements mediated by FNDC5 and irisin as lean individuals, such as increased expression of browning-related genes in proliferating cells and enhanced energy expenditure.

Numerous studies on rodents have consistently demonstrated that chronic exercise activity can stimulate bWAT through the activation of the sympathetic system (7, 16) and the secretion of myokines (6, 17). In lean rodents, this exercise-induced activation of bWAT is characterized by increased expression of markers such as peroxisome proliferator-activated receptor-gamma co-activator 1-alpha (PGC1α), FNDC5, and UCP1 (6, 18-20).

However, there are still some conflicting findings regarding the effects of physical activity on bWAT in obese individuals. While Li et al. observed an increase in markers related to bWAT following exercise in obesity conditions (8, 10, 21-23), others, such as Guilford et al., have reported a negative (12, 13) or neutral relationship (24, 25). Moreover, chronic cold exposure can induce sympathetic stimulation and a thermic response in white adipose tissue (WAT). The interaction between norepinephrine and β3 receptors on the membrane of white adipocytes triggers a cascade of events, leading to the overexpression of UCP1 and other thermogenic proteins (2). Therefore, cold exposure is considered one of the effective factors in stimulating bWAT and promoting thermogenesis in healthy metabolic conditions (7, 26, 27).

Investigating the relationship between cold exposure, particularly cold water, and its impact on obesity conditions can provide valuable insights, given the limited information available. The current study aims to assess the effects of shallow cold water, which has not been extensively studied before. Using cold water instead of cold weather allows for more precise control over temperature and other influential factors. Additionally, to accurately evaluate the impact of cold exposure without the confounding factor of swimming, a water depth of 2 - 4 cm was chosen (28), as swimming itself can affect bWAT. Furthermore, the existence of conflicting findings regarding the effects of obesity and physical activity led us to examine their combined effects on bWAT. While numerous studies have investigated the effects of various stimuli on bWAT and BAT activation, the focus has primarily been on individual stimuli, with less attention given to the combined effects of multiple stimuli.

## 2. Objectives

This study incorporated a combined intervention involving both exercise activity and cold water exposure to elucidate the effects of exercise and cold on obese rats.

## 3. Methods

### 3.1. Animal Subjects

A total of 25 male Wistar rats, aged 10 to 11 weeks and weighing 230 to 240 grams, were procured from the Mashhad Faculty of Medical Sciences. Subsequently, they were transferred to the animal laboratory at Ferdowsi University of Mashhad, specifically the Faculty of Sport Sciences. All experimental procedures were approved by the Research Ethics Committee of Ferdowsi University of Mashhad, with the ethical clearance code: IR.UM.REC.1401.124.

### 3.2. Acclimatization to the Laboratory Setting

The rats were housed individually in polycarbonate cages measuring 20 × 27 × 47 cm for a duration of one week. They were maintained under standardized conditions with a 12-hour light-dark cycle, a temperature of 23 ± 1°C, and a relative humidity of 50 ± 3 percent. Adequate water and standard food were made freely accessible. The daily intake of food and water was monitored throughout the study.

### 3.3. Induction of Obesity Using a High-Fat Diet

Following the familiarization period, the rats were fed a high-fat diet (HFD) devised by the researcher, which was composed of 47.3% lipids, for approximately four weeks. To confirm the induction of obesity, Lee’s index was calculated for all rats at the end of the obesity induction period. The Lee Index is defined as the cube root of body weight (g) divided by the naso-anal length (mm). Lee Index values above 310 g were considered indicative of obesity in rats (29). Body weight was measured using a digital scale with a precision of ± 0.01 g, and the naso-anal length was measured using a calibrated measuring tool. Measurements were performed under anesthesia induced by an intraperitoneal injection of ketamine (30 - 50 mg/kg) and xylazine (3 - 5 mg/kg). Rats with a Lee Index above the established threshold for obesity were categorized as obese. The use of anesthesia ensured accurate measurements while minimizing stress to the animals.

**Figure 1.**
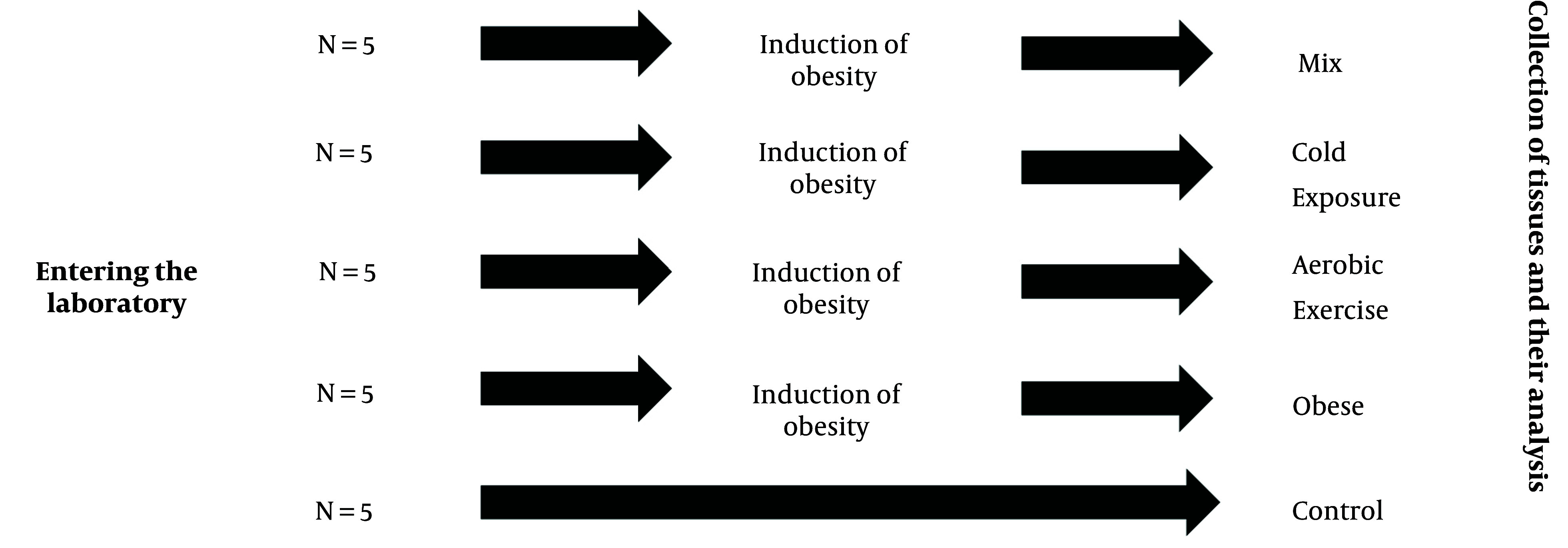
Grouping of rats

Once the rats met the obesity criteria, the next phase of the research involved shifting all rats to a healthy diet. At this point, the rats were divided into five distinct groups, each comprising 5 rats: (1) Control (C), (2) obesity (O), (3) obesity with cold water exposure (OC), (4) obesity with aerobic exercise (OE), and (5) obesity with cold water exposure and aerobic exercise (OCE). The arrangement of the rat groupings is depicted in Figure 1. Simple randomization was performed using the lottery method.

### 3.4. Protocol for Cold Water Exposure and Aerobic Exercise Intervention

After a week of acclimatization to the treadmill (consisting of five days at a speed of 10 m/min for 15 minutes), the exercise interventions for the OE and OCE groups were implemented using a specialized rodent treadmill, as outlined in Table 1. Each session included a five-minute warm-up and cool-down period, both performed at a pace of 10 meters per minute (30).

**Table 1.** Aerobic Exercise Protocol

Parameter	Week					
	1	2	3	4	5	6
**Speed (m/min)**	15	15	20	20	25	25
**Duration (min)**	30	40	40	50	50	60
**Distance (m)**	400	550	700	900	1100	1350

The procedure for the cold-water exposure intervention was conducted according to the guidelines specified in Table 2, for the OC and OCE groups (31, 32).

**Table 2.** Cold Water Exposure Protocol

Parameter	Week					
	1	2	3	4	5	6
**Temperature (°C)**	18	18	16	16	14	14
**Duration (min)**	30	40	40	50	50	60
**Water depth (cm)**	2	2	3	3	4	4

In the OCE group, both the OC and OE protocols were carried out within each session; however, the order in which these protocols were performed alternated daily. The interventions for the OC, OE, and OCE groups were carried out over a span of six weeks, with five sessions scheduled per week.

### 3.5. Collection of Samples

To minimize the stress response induced by the exercise session, the rats were anesthetized 48 hours after the last exercise session and 12 hours following fasting. An intraperitoneal injection of ketamine (30 to 50 mg per kilogram of body weight) and xylazine (3 to 5 mg per kilogram of body weight) was administered to induce anesthesia. The effectiveness of the anesthesia was confirmed by the absence of movement and lack of response to stimuli, such as pinching the toe. After anesthesia was achieved, a five to six-centimeter incision was made in the abdominal area to allow for the extraction of the soleus muscle and subcutaneous fat. These samples were then rinsed with physiological serum, placed in 1.5 mL microtubes, and transported to Ghaem Hospital in Mashhad for genetic testing. Following tissue extraction, the remaining carcasses were stored in a freezer at a temperature below freezing point. Ultimately, at the conclusion of the study, the carcasses were incinerated in the incinerator affiliated with the Faculty of Veterinary Medicine at Ferdowsi University of Mashhad.

### 3.6. RNA Extraction and RT-PCR Analysis of Soleus Muscle and Subcutaneous Fat

Fibronectin type-III domain containing 5 gene expression from soleus muscle tissue, along with FNDC5 and UCP1 expression from subcutaneous fat, was assessed using the RT-PCR method. RNA extraction was performed using the Favorgen^®^ mRNA extraction kit (Cat no: FATRS 050, Taiwan) according to the manufacturer’s instructions. To enable successful PCR amplification, the RNA was first converted into complementary DNA (cDNA). For the synthesis of the initial cDNA strand, two micrograms of mRNA from each sample was used. The cDNA synthesis was facilitated by the Addbio cDNA synthesis kit (Cat no: 22701, Korea), according to the manufacturer’s instructions. The synthesized cDNA was then used for RT-PCR analysis.

### 3.7. Real-time PCR Technique

To quantify the expression levels of FNDC5 and UCP1 genes, the real-time PCR technique was employed, using SYBR Green as the detection method. The primer sequences, as outlined in Table 3, were designed based on gene information obtained from the NCBI Gene Bank. The temperature profile for the RT-PCR process included initial denaturation at 95°C for 10 minutes, followed by denaturation at 90°C for 15 seconds, and annealing at 60°C for one minute, with a total of 40 cycles. The expression levels of FNDC5 and UCP1 genes were evaluated using the 2^-ΔΔCt^ method. After RNA extraction, spectrophotometry and agarose gel electrophoresis were used to assess the quality and quantity of the extracted RNA. It is noteworthy that GAPDH was used as a reference/control gene during the analysis.

**Table 3.** Primer Sequences

Gene	Forward 5'-3'	Reverse 5'-3'	Product Length
**FNDC5 NM_001171940.2**	CTTCATGTGGGCAGGTGTCAT	ATTGGGCTCGTTGTCCTTGAT	212
**UCP1 NM_009463.3**	GCTCCTCCACAAATAGCCCTG	CGGAAGTTGTCGAACTCACCA	307
**GAPDH NM_001256799.3**	CTCTCTGCTCCTCCCTGTTC	CGATACGGCCAAATCCGTTC	333

### 3.8. Statistical Analysis

Data analysis was performed using SPSS software, version 16. The comparison of average ranks among the groups was carried out using the Kruskal-Wallis test. Subsequently, the U-Mann Whitney post hoc test was applied to assess pairwise differences between the groups. The hypotheses were evaluated at a significance threshold of P < 0.05.

## 4. Results

### 4.1. Lee Index

As shown in Table 4, Lee Index values above 310 g were considered an indicator of obesity in rats (29).

**Table 4.** The Mean Weight and Lee Index of the Groups at the End of the Obesity Induction Period

Group	Weight	Lee Index
**Control**	276	301
**Obese**	322.7	315
**Aerobic exercise**	321	313
**Cold exposure**	328.2	316
**Mix**	329.8	316

As shown in Table 5, within-group weight changes during the 6 weeks of training intervention were significant for all obese groups. However, between-group weight changes were not significant.

**Table 5.** Weight Changes Within and Between Groups During 6 Weeks of Exercise Intervention in Obese Groups

Group	F	P-Value
**Within-group**	130.277	0.000
**Between-group**	1.058	0.403

**Figure 2.**
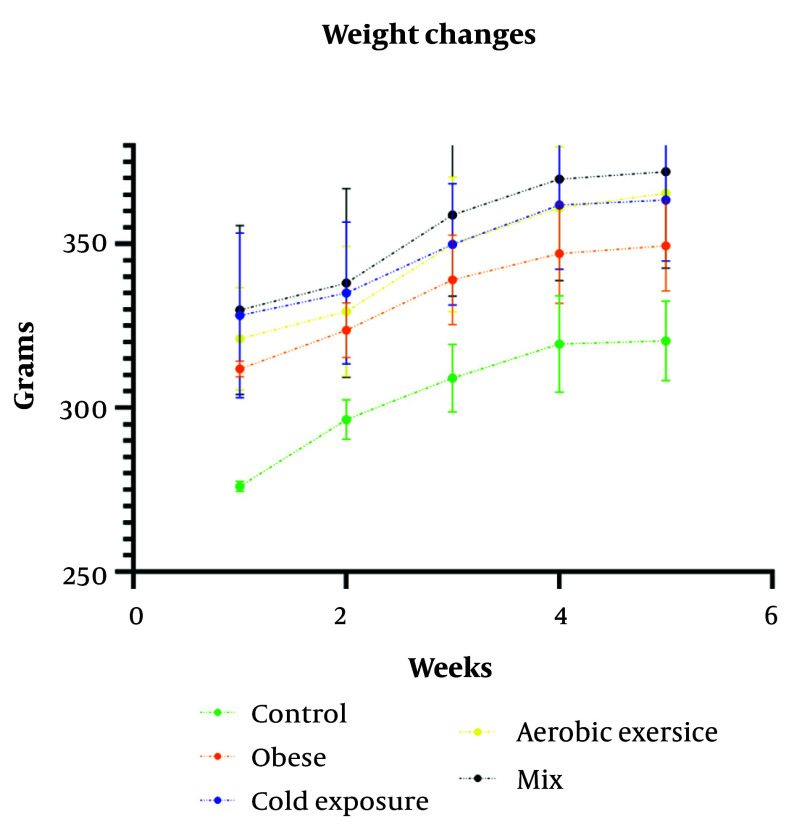
Weight changes of rats during the implementation of the protocols; the mean is indicated by the gray color, and the standard deviation is represented by the specific color of each group.

The changes in the groups' weights throughout the execution of the protocols and after discontinuing the high-fat diet are shown in Figure 2. The weight increase in all groups was consistently monitored over the course of the six-week intervention period.

### 4.2. Fibronectin Type-III Domain Containing 5 in Soleus Muscle in Male Wistar Rats

**Figure 3.**
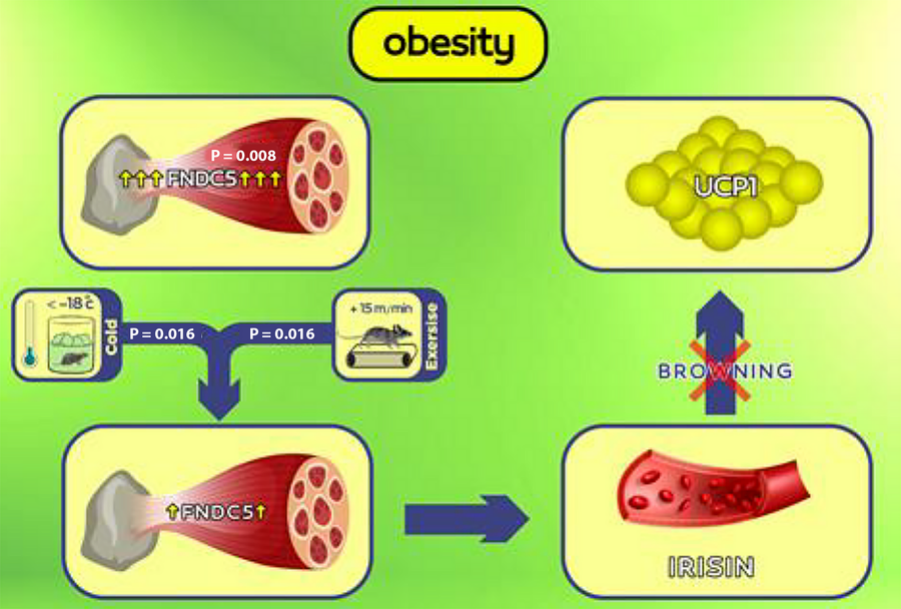
Graphical abstract: In the case of obesity, overexpression of the muscle fibronectin type-III domain containing 5 (FNDC5) gene can lead to a pathological condition where the increase in muscle FNDC5 gene expression is not aligned with the increase in fat uncoupling protein 1 (UCP1) gene expression and thermogenesis. Six weeks of exposure to cold and aerobic exercise can reduce muscle FNDC5 gene expression.

Figure 3 illustrates the impact of obesity on the significant elevation of FNDC5 gene expression (P = 0.008). The six-week period involving cold water exposure (P = 0.016) or aerobic exercise (P = 0.016) notably reduced FNDC5 gene expression, with no significant difference between the two methods (P = 0.83). However, the combined intervention involving both cold water exposure and aerobic exercise did not exhibit a significant effect (P = 0.75).

### 4.3. Uncoupling Protein 1 in Subcutaneous Fat in Male Wistar Rats

Obesity (P = 0.074), cold water exposure (P = 0.59), aerobic exercise (P = 0.347), and the combined intervention (P = 0.91) did not have any significant impact on UCP1 levels.

### 4.4. Fibronectin Type-III Domain Containing 5 in Subcutaneous Fat in Male Wistar Rats

The results of the Kruskal-Wallis test indicated that none of the interventions had a significant effect on FNDC5 gene expression within subcutaneous fat (Chi-square = 5.835, df = 4, P = 0.212). Descriptive statistics related to the expression of FNDC5 and UCP1 genes are presented in Table 6. 

**Table 6.** Descriptive Statistics Related to the Expression of Fibronectin Type-III Domain Containing 5 and Uncoupling Protein 1 Genes in the Groups

Variables and Groups	Mean ± SD (Median)
**Muscle FNDC5 (relative expression)**	
Control	0.99 ± 0.00 (0.99)
Obese	5.41 ± 2.40 (4.50)
Cold exposure	1.91 ± 0.96 (2.00)
Aerobic exercise	1.76 ± 1.31 (2.02)
Mix	4.82 ± 2.14 (5.20)
**UCP1 (relative expression)**	
Control	0.99 ± 0.00 (0.99)
Obese	3.26 ± 2.31 (2.26)
Cold exposure	3.40 ± 1.65 (2.92)
Aerobic exercise	1.97 ± 1.35 (1.00)
Mix	2.78 ± 1.94 (2.84)
**Fat FNDC5 (relative expression)**	
Control	0.99 ± 0.00 (0.99)
Obese	1.97 ± 1.79 (0.87)
Cold exposure	4.34 ± 6.61 (1.97)
Aerobic exercise	1.88 ± 1.83 (1.02)
Mix	0.53 ± 0.29 (0.43)

Abbreviations: FNDC5, fibronectin type-III domain containing 5; UCP1, uncoupling protein 1.

## 5. Discussion

This study demonstrated that obesity significantly increased the expression of the FNDC5 gene. Over a span of six weeks, both aerobic exercise and cold water exposure led to a significant reduction in its expression, although the combination of both interventions did not have a notable impact. In contrast, none of the interventions targeting obesity—whether a six-week course of aerobic exercise, cold water exposure, or their combination—resulted in a significant change in UCP1 gene expression.

Previous studies conducted by Badawy et al. and Wu et al. did not observe significant changes in FNDC5 content in rats fed consistently with high-fat and high-carbohydrate diets compared to those on standard diets (14, 15). However, contrasting findings were reported by Kazemi Nasab et al. and Guilford et al., who found higher levels of muscle PGC1α and FNDC5 in mice subjected to high-fat diets compared to those on low-fat diets. In line with our findings, a positive correlation between muscle FNDC5 expression and body fat was also established (12, 13).

The increase in FNDC5 expression within skeletal muscles may aim to promote energy expenditure and act as a compensatory mechanism against weight gain associated with a high-fat diet. However, this elevation, combined with potential chronic increases in irisin levels during obesity, could potentially lead to fat tissue resistance to this hormone. Numerous studies have indeed identified the highest levels of irisin in obese individuals (33, 34), with FNDC5 and irisin levels decreasing following weight loss (11). This rise in irisin levels in obesity might serve as a feedback mechanism for metabolic regulation. Additionally, obese individuals may develop resistance to irisin similar to the resistance observed with insulin and leptin (35). Moreover, since FNDC5 is recognized as a heat-generating factor (36), with heat being its final product instead of ATP, it is plausible to suggest that increased FNDC5 expression under conditions of excess energy intake may not be far-fetched (37). Conversely, studies have demonstrated that energy restriction leads to a decrease in heat generation (38).

Nevertheless, the precise mechanism of FNDC5 and irisin action in obesity requires further exploration, as it remains unclear whether the heightened expression of these genes following obesity contributes to enhanced energy expenditure or if this elevation itself functions as a pathological factor. Thus, obese individuals may not experience the same metabolic improvements mediated by FNDC5 and irisin—such as increased expression of browning-related genes in mature adipocytes and enhanced energy expenditure—that lean individuals do (39).

This study aligns with Eslami et al., who noted no significant elevation in UCP1 gene expression in subcutaneous fat (40). Uncoupling protein 1 serves as a marker of thermogenesis in mitochondria and exhibits lipolytic and oxidative properties. Its role in metabolizing substrates and converting heat into ATP is also noteworthy (41). These results indicate that, in obese rats, despite the increased expression of the FNDC5 gene, the process of browning white fat is reduced, while the storage of fat as white adipocytes is increased. In the context of obese mice, our results show an increase in body weight across all groups, including the obese group, throughout the intervention protocols (Figure 2). Aerobic exercise has been shown to increase muscle mass to some extent, which could contribute to weight gain in the exercised groups (42).

Li et al. revealed that a high-fat diet led to decreased muscle FNDC5 and UCP1 levels within the adipose tissue of mice. In contrast, exercise, diet control, or a combination of both resulted in significantly reduced body weight, white adipose mass, and lipid ratios in obese mice. Notably, our findings highlight the continuous weight increase observed across all groups. These outcomes are consistent with the changes in UCP1 identified in the aforementioned studies (10).

The time required for obesity to manifest ranges from 8 days to 27 weeks. Obesity-related characteristics, such as increased glucose intolerance, become more evident after prolonged exposure to obesity-promoting diets. While some studies suggest that the seventh week marks a significant increase in weight gain (43), others advocate for a 10- to 12-week duration to fully consolidate phenotypic and metabolic attributes of obesity (43). In our study, obesity was induced over a brief four-week period, which contrasts with Li et al. and Yang et al., where the induction lasted 10 and 11 weeks, respectively (10, 44). Additionally, all rodents in Li et al.'s study maintained a high-fat diet throughout, while our intervention period of six weeks involved rats on a healthy diet (10).

Across most studies, FNDC5, irisin, and UCP1 proteins have been key parameters for evaluating bWAT. However, in our study, we assessed the expression of FNDC5 and UCP1 genes. This distinction could be attributed to post-transcriptional and post-translational modifications. For example, despite the overexpression of the FNDC5 gene, its protein may not be synthesized, or its cleavage into irisin may be reduced under obesity conditions.

Inflammation emerges as another factor that could contribute to these discrepancies. Mature adipocytes are known to secrete pro-inflammatory cytokines, which contribute to systemic inflammation and complications in obese individuals (45). In light of this, some studies propose that impaired BAT/Beige thermogenesis during obesity may result from the propagation of inflammation (46). It is plausible that, at the onset of obesity, the body increases heat generation as a strategy to counteract further fat accumulation. However, as obesity progresses and inflammation-related disorders develop, bWAT becomes suppressed. Therefore, a comprehensive study encompassing various degrees of obesity, inflammation spread, and bWAT assessment could potentially clarify these contradictions.

This study demonstrated a notable decrease in FNDC5 expression in the soleus muscle of obese rats after six weeks of cold water exposure. The resulting heat generation related to shivering thermogenesis might explain the reduced demand for non-shivering thermogenesis and the corresponding decline in FNDC5 expression. These findings contrast with those of Lee et al. and Reisi and Mohammadnia (7, 32). The discrepancy may be attributed to protocol differences, as our design prevented full immersion of the rats due to the 2 - 4 cm water depth, whereas Badawi et al. and Cho et al. observed increased FNDC5, irisin, and UCP1 following acute and chronic swimming in (14, 20). Further studies have supported these observations (8, 28).

Lee et al. found that cold exposure increased irisin secretion, with colder temperatures leading to higher irisin production (7). However, Lee et al.'s study involved a single session with healthy subjects, intermittently reducing the temperature until it reached 12°C (7). A prolonged response to cold might differ from a single-session exposure. For example, long-term cold exposure studies have reported no similar increase (47, 48). Additionally, the healthy metabolic state of the subjects in Lee et al.'s and Reisi and Mohammadnia's studies may have influenced the outcomes, as Vijgen et al. identified weakened thermogenesis due to cold exposure during obesity (49). This study indicated a significant reduction in FNDC5 expression in the soleus muscle of obese rats after six weeks of cold water exposure, which may represent a compensatory mechanism against the heightened FNDC5 expression associated with obesity.

In our study, six weeks of cold water exposure did not lead to any changes in UCP1 gene expression in subcutaneous fat. Although rodent studies have highlighted the positive impact of cold exposure on bWAT, human experimental evidence remains inconclusive. Some studies have shown that long-term cold exposure increases PGC1α, UCP1, and mitochondrial activity, all indicative of beige cell characteristics (48). However, other research did not report significant changes in UCP1 or other bWAT markers in healthy human subcutaneous fat following 10 days of cold acclimation (49), which aligns with further studies (47). Variations in cold intensity may contribute to these conflicting results, as rodents are typically exposed to temperatures around 4°C, which is impractical for humans. Temperatures between 14 - 18°C likely proved insufficient to induce changes in UCP1 gene expression, suggesting the need for lower temperatures (50). Additionally, the hypothesis of irisin resistance, as discussed earlier, could be a contributing factor.

The present study did not reveal significant changes in UCP1 gene expression following the OE intervention. However, FNDC5 gene expression experienced a significant decline in this group. This is consistent with Davis et al., where markers of bWAT, including UCP1, showed no change in inguinal or epididymal fat after 15 weeks of running exercise (24). Aldiss et al. observed no change in UCP1 mRNA within inguinal subcutaneous fat after four weeks of swimming exercise (25). Additionally, Ramos et al. found no impact on mitochondrial proteins, including UCP1, after eight weeks of treadmill exercise (18 to 25 m/min for 30 to 60 minutes with a 10% incline) (51). Conversely, Guilford et al. noted a significant reduction in UCP1 in epididymal fat tissue after four weeks of running wheel activity. Moreover, exercise did not significantly alter FNDC5 or PGC1α protein levels in the skeletal muscle of obese mice (12). These variations could be attributed to methodological differences, such as the rodents' metabolic condition, the type, intensity, and duration of exercise. As such, Wu et al. demonstrated that, under obesity conditions, the effect of exercise-induced browning in inguinal subcutaneous fat tissue is reduced (15). Furthermore, our exercise intensity ranged from approximately 50 - 70% of maximal oxygen consumption (52). In this context, Tanimura et al. reported no change in UCP1 gene expression in inguinal subcutaneous fat following low-intensity exercise, in contrast to high-intensity exercise (53).

However, despite irisin being recognized as both a myokine and adipokine (54), the exact nature of bWAT regulation—whether it operates through an endocrine, autocrine, or mixed mechanism—remains uncertain. Our findings in Table 6 show no significant changes in FNDC5 gene expression or UCP1 gene expression in subcutaneous fat for any of the interventions. This divergence could be due to post-transcriptional and post-translational modifications. Contrary to expectations, Lee et al. found that cold exposure was associated with decreased irisin secretion, with more irisin being produced at lower temperatures (7). However, Lee et al.'s study was conducted in healthy individuals over a single session, during which the temperature gradually decreased to 12°C (7). Prolonged cold exposure might yield different results compared to a single session. Additionally, since Lee et al. and Reisi and Mohammadnia studied healthy subjects (7, 32), their observations could be attributed to the healthy metabolic state of these individuals, as indicated by Vijgen et al., who identified weakened cold-induced thermogenesis during obesity (49). In our study, a significant reduction in FNDC5 expression was observed within the soleus muscle of obese rats after six weeks of cold water exposure. This decrease may represent a compensatory mechanism against the elevated FNDC5 expression typically seen in obesity.

Lastly, a combined intervention of aerobic exercise and cold water exposure was employed to investigate their effects on obese rats. However, the results did not show a significant change in FNDC5 gene expression within the soleus muscle or UCP1 gene expression in subcutaneous fat between the OCE and O groups.

In Javadifar et al.'s study, obese male rats with type 2 diabetes underwent various interventions, including resistance exercises at both normal and cold temperatures, as well as endurance exercises at normal and cold temperatures. Meanwhile, control groups were exposed to normal and cold temperatures without physical activity. Interestingly, no significant differences in irisin levels were observed between the exercise and control groups at both temperatures, although irisin levels were slightly higher in the cold group (48). Ozbay et al. had 32 healthy men participate in 40-minute aerobic running sessions four days a week, at -5 to 5°C (16 participants) and 21 - 25°C (16 participants). After 18 weeks, irisin levels remained unchanged in the cold and exercise group but significantly decreased in the other group (55). The differing thermal conditions between exercise and cold exposure might contribute to the disparity in their effects. Cold exposure triggers both shivering and non-shivering thermogenesis, while exercise is inherently thermogenic, making it unlikely to further enhance thermogenic activity. This could explain the absence of significant effects on bWAT factors in our study, as well as in Javadifar et al.'s and Ozbay et al.'s studies (48, 55).

Furthermore, the OCE group simultaneously underwent both cold water exposure and aerobic exercise protocols during each session. The substantial activity volume in this group, with relatively high energy expenditure, could potentially induce energy restriction. As NST primarily focuses on heat production and energy loss, it is plausible that reducing thermogenesis serves as a protective mechanism in this scenario. Afshari et al. observed that high-volume aerobic exercise failed to elevate UCP1 expression in subcutaneous fat compared to moderate-volume exercise. The high energy expenditure in high-volume exercise might trigger the body's defense mechanisms against energy loss (56). This mechanism could be analogous to the conditions of energy restriction (38).

### 5.1. Limitations

This study has some limitations. Although gene expression plays a crucial role, the proteins produced by these genes are ultimately responsible for executing their functions. Due to financial constraints, this study only examined gene expression. Additionally, instead of considering body fat percentage, we used weight gain as the criterion for obesity induction.

In this study, the HFD used to induce obesity was based on a standard rodent chow diet with a specific percentage of fat. This approach was designed to meet baseline nutritional requirements for essential vitamins and minerals. However, we acknowledge that altering the composition of the diet's macronutrients could potentially affect the balance of micronutrients, leading to suboptimal intake of some vitamins and minerals.

While no signs of malnutrition were observed during the study, this limitation should be considered when interpreting the results. Future studies could incorporate fortified HFD formulations or conduct more detailed nutritional analyses to ensure the adequacy of all essential nutrients.

### 5.2. Conclusions

In conclusion, comprehensive findings on the combined impact of exercise and cold exposure are scarce, with considerable heterogeneity among the few studies conducted. Thus, further investigations comparing the effects of these two protocols could provide more insight into this matter.

## Data Availability

The dataset presented in the study is available on request from the corresponding author during submission or after publication.

## References

[1] Finkelstein EA, Khavjou OA, Thompson H, Trogdon JG, Pan L, Sherry B (2012). Obesity and severe obesity forecasts through 2030.. Am J Prev Med..

[2] Machado SA, Pasquarelli-do-Nascimento G, da Silva DS, Farias GR, de Oliveira Santos I, Baptista LB (2022). Browning of the white adipose tissue regulation: new insights into nutritional and metabolic relevance in health and diseases.. Nutr Metab (Lond)..

[3] The Commission for Thermal Physiology of the International Union of Physiological Sciences (2001). Glossary of terms for thermal physiology.. Jpn J Physiol..

[4] Blondin DP, Haman F (2018). Shivering and nonshivering thermogenesis in skeletal muscles.. Handb Clin Neurol..

[5] Peres Valgas da Silva C, Hernandez-Saavedra D, White JD, Stanford KI (2019). Cold and Exercise: Therapeutic Tools to Activate Brown Adipose Tissue and Combat Obesity.. Biology (Basel)..

[6] Bostrom P, Wu J, Jedrychowski MP, Korde A, Ye L, Lo JC (2012). A PGC1-alpha-dependent myokine that drives brown-fat-like development of white fat and thermogenesis.. Nature..

[7] Lee P, Linderman JD, Smith S, Brychta RJ, Wang J, Idelson C (2014). Irisin and FGF21 are cold-induced endocrine activators of brown fat function in humans.. Cell Metab..

[8] Lu Y, Li H, Shen S, Shen Z, Xu M, Yang C (2016). Swimming exercise increases serum irisin level and reduces body fat mass in high-fat-diet fed Wistar rats.. Lipids Health Dis..

[9] Mostafavian M, Abdi A, Mehrabani J, Barari A (2020). [Effect of Eight Weeks of Aerobic Progressive Training with Capsaicin on Changes in PGC-1α and UPC-1 Expression in Visceral Adipose Tissue of Obese Rats With Diet].. Complement Med J..

[10] Li J, Yi X, Li T, Yao T, Li D, Hu G (2022). Effects of exercise and dietary intervention on muscle, adipose tissue, and blood IRISIN levels in obese male mice and their relationship with the beigeization of white adipose tissue.. Endocr Connect..

[11] Huh JY, Panagiotou G, Mougios V, Brinkoetter M, Vamvini MT, Schneider BE (2012). FNDC5 and irisin in humans: I. Predictors of circulating concentrations in serum and plasma and II. mRNA expression and circulating concentrations in response to weight loss and exercise.. Metabolism..

[12] Guilford BL, Parson JC, Grote CW, Vick SN, Ryals JM, Wright DE (2017). Increased FNDC5 is associated with insulin resistance in high fat-fed mice.. Physiol Rep..

[13] Kazemi Nasab F, Marandi M, Ghaedi K, Esfarjani F, Nasr-Esfahani MH (2019). The Effect of Endurance Training and High-Fat Diet on PGC-1a/FNDC5/Irisin Pathway in Male C57BL/6 Mice.. Sports Physiol..

[14] Badawy E, El-laithy NA, Morsy SM, Ashour MN, Elias TR, Masoud MM (2020). Role of swimming on muscle PGC-1α, FNDC5 mRNA, and assessment of serum omentin, adropin, and irisin in high carbohydrate high fat (HCHF) diet induced obesity in rats.. Egypt J Med Hum Genet..

[15] Wu MV, Bikopoulos G, Hung S, Ceddia RB (2014). Thermogenic capacity is antagonistically regulated in classical brown and white subcutaneous fat depots by high fat diet and endurance training in rats: impact on whole-body energy expenditure.. J Biol Chem..

[16] Tsiloulis T, Watt MJ (2015). Exercise and the Regulation of Adipose Tissue Metabolism.. Prog Mol Biol Transl Sci..

[17] Dewal RS, Stanford KI (2019). Effects of exercise on brown and beige adipocytes.. Biochim Biophys Acta Mol Cell Biol Lipids..

[18] Lehnig AC, Dewal RS, Baer LA, Kitching KM, Munoz VR, Arts PJ (2019). Exercise Training Induces Depot-Specific Adaptations to White and Brown Adipose Tissue.. iScience..

[19] Dehghani M, Kargarfard M, Rabiee F, Nasr-Esfahani MH, Ghaedi K (2018). A comparative study on the effects of acute and chronic downhill running vs uphill running exercise on the RNA levels of the skeletal muscles PGC1-alpha, FNDC5 and the adipose UCP1 in BALB/c mice.. Gene..

[20] Cho E, Jeong DY, Kim JG, Lee S (2021). The Acute Effects of Swimming Exercise on PGC-1alpha-FNDC5/Irisin-UCP1 Expression in Male C57BL/6J Mice.. Metabolites..

[21] Schaalan MF, Ramadan BK, Abd Elwahab AH (2018). Synergistic effect of carnosine on browning of adipose tissue in exercised obese rats; a focus on circulating irisin levels.. J Cell Physiol..

[22] Bae JY (2018). Aerobic Exercise Increases Meteorin-Like Protein in Muscle and Adipose Tissue of Chronic High-Fat Diet-Induced Obese Mice.. Biomed Res Int..

[23] Barjaste Yazdi A, Matin Homaee H, Peeri M (2018). The Effect of Endurance Exercise and Adenosine Consumption on UCP-1 Gene Expression in the Visceral Adipose Tissue of Obese Male Rats.. Iran J Diabetes Obes..

[24] Davis RAH, Halbrooks JE, Watkins EE, Fisher G, Hunter GR, Nagy TR (2017). High-intensity interval training and calorie restriction promote remodeling of glucose and lipid metabolism in diet-induced obesity.. Am J Physiol Endocrinol Metab..

[25] Aldiss P, Lewis JE, Lupini I, Bloor I, Chavoshinejad R, Boocock DJ (2020). Exercise Training in Obese Rats Does Not Induce Browning at Thermoneutrality and Induces a Muscle-Like Signature in Brown Adipose Tissue.. Front Endocrinol (Lausanne)..

[26] Bal NC, Singh S, Reis FCG, Maurya SK, Pani S, Rowland LA (2017). Both brown adipose tissue and skeletal muscle thermogenesis processes are activated during mild to severe cold adaptation in mice.. J Biol Chem..

[27] Yu J, Zhang S, Cui L, Wang W, Na H, Zhu X (2015). Lipid droplet remodeling and interaction with mitochondria in mouse brown adipose tissue during cold treatment.. Biochim Biophys Acta..

[28] Yang XQ, Yuan H, Li J, Fan JJ, Jia SH, Kou XJ (2016). Swimming intervention mitigates HFD-induced obesity of rats through PGC-1alpha-irisin pathway.. Eur Rev Med Pharmacol Sci..

[29] Bastias-Perez M, Serra D, Herrero L (2020). Dietary Options for Rodents in the Study of Obesity.. Nutrients..

[30] Hung YH, Linden MA, Gordon A, Rector RS, Buhman KK (2015). Endurance exercise training programs intestinal lipid metabolism in a rat model of obesity and type 2 diabetes.. Physiol Rep..

[31] da Silva JT, Cella PS, Testa MTJ, Perandini LA, Festuccia WT, Deminice R (2020). Mild-cold water swimming does not exacerbate white adipose tissue browning and brown adipose tissue activation in mice.. J Physiol Biochem..

[32] Reisi J, Mohammadnia AM (2016). Effect of cold and moderate water immersion during resistance exercise on plasma irisin protein response in rats.. Archive of SID..

[33] Chen JQ, Fang LJ, Song KX, Wang XC, Huang YY, Chai SY (2016). Serum Irisin Level is Higher and Related with Insulin in Acanthosis Nigricans-related Obesity.. Exp Clin Endocrinol Diabetes..

[34] Palacios-Gonzalez B, Vadillo-Ortega F, Polo-Oteyza E, Sanchez T, Ancira-Moreno M, Romero-Hidalgo S (2015). Irisin levels before and after physical activity among school-age children with different BMI: a direct relation with leptin.. Obesity (Silver Spring)..

[35] Luo Y, Qiao X, Xu L, Huang G (2022). Irisin: circulating levels in serum and its relation to gonadal axis.. Endocrine..

[36] Rabiee F, Lachinani L, Ghaedi S, Nasr-Esfahani MH, Megraw TL, Ghaedi K (2020). New insights into the cellular activities of Fndc5/Irisin and its signaling pathways.. Cell Biosci..

[37] Tremblay A, Despres JP, Theriault G, Fournier G, Bouchard C (1992). Overfeeding and energy expenditure in humans.. Am J Clin Nutr..

[38] Rothwell NJ, Stock MJ (1982). Effect of chronic food restriction on energy balance, thermogenic capacity, and brown-adipose-tissue activity in the rat.. Biosci Rep..

[39] Polyzos SA, Kountouras J, Shields K, Mantzoros CS (2013). Irisin: a renaissance in metabolism?. Metabolism..

[40] Eslami Z, Mohammadnajad PanahKandi Y, Sharifian S, Eghbal Moghanlou A, Sheikh SR, Mirghani SJ (2021). Evaluation of the effect of aerobic exercise on UCP1 and MAPK p38 heat factor gene expression in subcutaneous adipose tissue in male Wistar rats fed a high-fat diet.. Feyz Med Sci J..

[41] Hirschenson J, Melgar-Bermudez E, Mailloux RJ (2022). The Uncoupling Proteins: A Systematic Review on the Mechanism Used in the Prevention of Oxidative Stress.. Antioxidants (Basel)..

[42] Franca GO, Frantz EDC, Magliano DC, Bargut TCL, Sepulveda-Fragoso V, Silvares RR (2020). Effects of short-term high-intensity interval and continuous exercise training on body composition and cardiac function in obese sarcopenic rats.. Life Sci..

[43] de Moura EM, Dos Reis SA, da Conceicao LL, Sediyama C, Pereira SS, de Oliveira LL (2021). Diet-induced obesity in animal models: points to consider and influence on metabolic markers.. Diabetol Metab Syndr..

[44] Yang B, Yu Q, Chang B, Guo Q, Xu S, Yi X (2021). MOTS-c interacts synergistically with exercise intervention to regulate PGC-1alpha expression, attenuate insulin resistance and enhance glucose metabolism in mice via AMPK signaling pathway.. Biochim Biophys Acta Mol Basis Dis..

[45] Kawai T, Autieri MV, Scalia R (2021). Adipose tissue inflammation and metabolic dysfunction in obesity.. Am J Physiol Cell Physiol..

[46] Omran F, Christian M (2020). Inflammatory Signaling and Brown Fat Activity.. Front Endocrinol (Lausanne)..

[47] Bubak MP, Heesch MWS, Shute RJ, Dinan NE, Laursen TL, L. A. Salle DT (2017). Irisin and Fibronectin Type III Domain-Containing 5 Responses to Exercise in Different Environmental Conditions.. Int J Exerc Sci..

[48] Javadifar K, Gaeini AA, Haddadi R, Ravasi AA (2021). Interactive effect of endurance exercise, resistance exercise, and cold weather on irisin changes in diabetic male rats.. J Basic Res Med Sci..

[49] Vijgen GH, Bouvy ND, Teule GJ, Brans B, Schrauwen P, van Marken Lichtenbelt WD (2011). Brown adipose tissue in morbidly obese subjects.. PLoS One..

[50] van der Lans AA, Hoeks J, Brans B, Vijgen GH, Visser MG, Vosselman MJ (2013). Cold acclimation recruits human brown fat and increases nonshivering thermogenesis.. J Clin Invest..

[51] Ramos SV, Turnbull PC, MacPherson RE (2016). Adipose tissue depot specific differences of PLIN protein content in endurance trained rats.. Adipocyte..

[52] Qin F, Dong Y, Wang S, Xu M, Wang Z, Qu C (2020). Maximum oxygen consumption and quantification of exercise intensity in untrained male Wistar rats.. Sci Rep..

[53] Tanimura R, Kobayashi L, Shirai T, Takemasa T (2022). Effects of exercise intensity on white adipose tissue browning and its regulatory signals in mice.. Physiol Rep..

[54] Roca-Rivada A, Castelao C, Senin LL, Landrove MO, Baltar J, Belen Crujeiras A (2013). FNDC5/irisin is not only a myokine but also an adipokine.. PLoS One..

[55] Ozbay S, Ulupinar S, Sebin E, Altinkaynak K (2020). Acute and chronic effects of aerobic exercise on serum irisin, adropin, and cholesterol levels in the winter season: Indoor training versus outdoor training.. Chin J Physiol..

[56] Afshari S, Mohammad-Amoli M, Daneshyar S (2017). Comparison of Moderate and High Volume Aerobic Training on Gene Expression of Uncoupling Protein 1 (UCP-1) in Subcutaneous White Adipose Tissue of Wistar Rats.. Iran J Endocrinol Metab..

